# Predicting risk for trauma patients using static and dynamic information from the MIMIC III database

**DOI:** 10.1371/journal.pone.0262523

**Published:** 2022-01-19

**Authors:** Evan J. Tsiklidis, Talid Sinno, Scott L. Diamond

**Affiliations:** Department of Chemical and Biomolecular Engineering, Institute for Medicine and Engineering, University of Pennsylvania, Philadelphia, PA, United States of America; Texas A&M University, UNITED STATES

## Abstract

Risk quantification algorithms in the ICU can provide (1) an early alert to the clinician that a patient is at extreme risk and (2) help manage limited resources efficiently or remotely. With electronic health records, large data sets allow the training of predictive models to quantify patient risk. A gradient boosting classifier was trained to predict high-risk and low-risk trauma patients, where patients were labeled high-risk if they expired within the next 10 hours or within the last 10% of their ICU stay duration. The MIMIC-III database was filtered to extract 5,400 trauma patient records (526 non-survivors) each of which contained 5 static variables (age, gender, etc.) and 28 dynamic variables (e.g., vital signs and metabolic panel). Training data was also extracted from the dynamic variables using a 3-hour moving time window whereby each window was treated as a unique patient-time fragment. We extracted the mean, standard deviation, and skew from each of these 3-hour fragments and included them as inputs for training. Additionally, a survival metric upon admission was calculated for each patient using a previously developed National Trauma Data Bank (NTDB)-trained gradient booster model. The final model was able to distinguish between high-risk and low-risk patients to an AUROC of 92.9%, defined as the area under the receiver operator characteristic curve. Importantly, the dynamic survival probability plots for patients who die appear considerably different from those who survive, an example of reducing the high dimensionality of the patient record to a single trauma trajectory.

## 1. Introduction

Trauma is the leading cause of death in the United States for people under the age of 46 and the leading cause of overall expected years of life lost [1]. Since modern intensive care units (ICU) monitor patients continuously, the data-rich environment can be used to predict mortality, time-dependent risk, and provide opportunities for data science and machine learning [2,3]. Due to the high patient-to-patient variability, a data-driven approach is a reasonable way of predicting patient outcomes as patient-scale mechanistic models developed from first principles are highly challenging and likely injury-specific [4–6]. Furthermore, time-series classification has already been successfully implemented in the field of medical diagnosis suggesting its potential utility in the context of trauma [7]. The Medical Information Mart for Intensive Care (MIMIC-III) database is one of the first initiatives for not only the frequent collection of clinical patient data, but also the public dissemination of this de-identified data to be used by researchers around the world. This database is now the gold standard for publicly available time-stamped patient data, consisting of a very large patient population (> 40,000 patients) and time-stamped records of every clinical event.

To date, there has been considerable work aimed at predicting ICU patient outcomes using existing databases. Alistair et al. predicted mortality to an area under the receiver operator characteristic curve (AUROC) of 92.4% using features extracted from the first 24 hour of a patient’s stay in the ICU [8]. Harutyunyan et al. predicted mortality within 24 hours to an AUROC of 91.1%, while simultaneously predicting average length of stay, an additional important variable for quantifying the efficacy of the ICU [9]. In an earlier study, our group predicted mortality in the National Trauma Data Bank (NTDB) to an AUROC of 91.8%, using only 8 static data points [age, gender, respiratory rate, heart rate, systolic blood pressure, Glasgow coma score, temperature, oxygen saturation] obtained upon admission into the hospital or ambulance, but not including data collected in the ICU [10]. In addition to mortality analysis, length-of-stay prediction has been the focus of many machine learning groups as well, as it is a useful measure for managing hospital resources, improving outcomes, and increasing efficiency [11,12]. Liu et al. published a detailed review on machine learning for trauma patients [13]. There have also been physics-based models constructed to model trauma. Ursino et al., developed a system of ordinary differential equations to describe the circulatory system as a closed loop with feedback, which has been extended to simulate traumatic bleeding [5,14]. While able to simulate blood loss patterns, these types of models are difficult to connect a specific injury to an outcome [4,15–17]. Hirshberg et al., also developed a model to evaluate the impact of the transfusion of blood products on dilutional coagulopathy and found that resuscitation with more than 5 units of red blood cells would lead to coagulopathy [18].

In this paper, we use the MIMIC-III ICU database to predict risk of death in trauma patients and whether a patient’s health will begin to rapidly decline (analogously, a rapid rise in risk). We pose this problem as a time-series classification problem where the input is a fixed-length window of patient properties (both dynamic and static) with a 1-hour step size. Each patient 3-hour window is regarded as an individual patient-time fragment that is used for training or evaluating a model. The goal of this work is to develop a model that can continuously assess and predict patient mortality probability (a metric for quantifying patient risk) as data becomes available in real time.

## 2. Methods

### 2.1 Patient dataset

**Fig 1:**
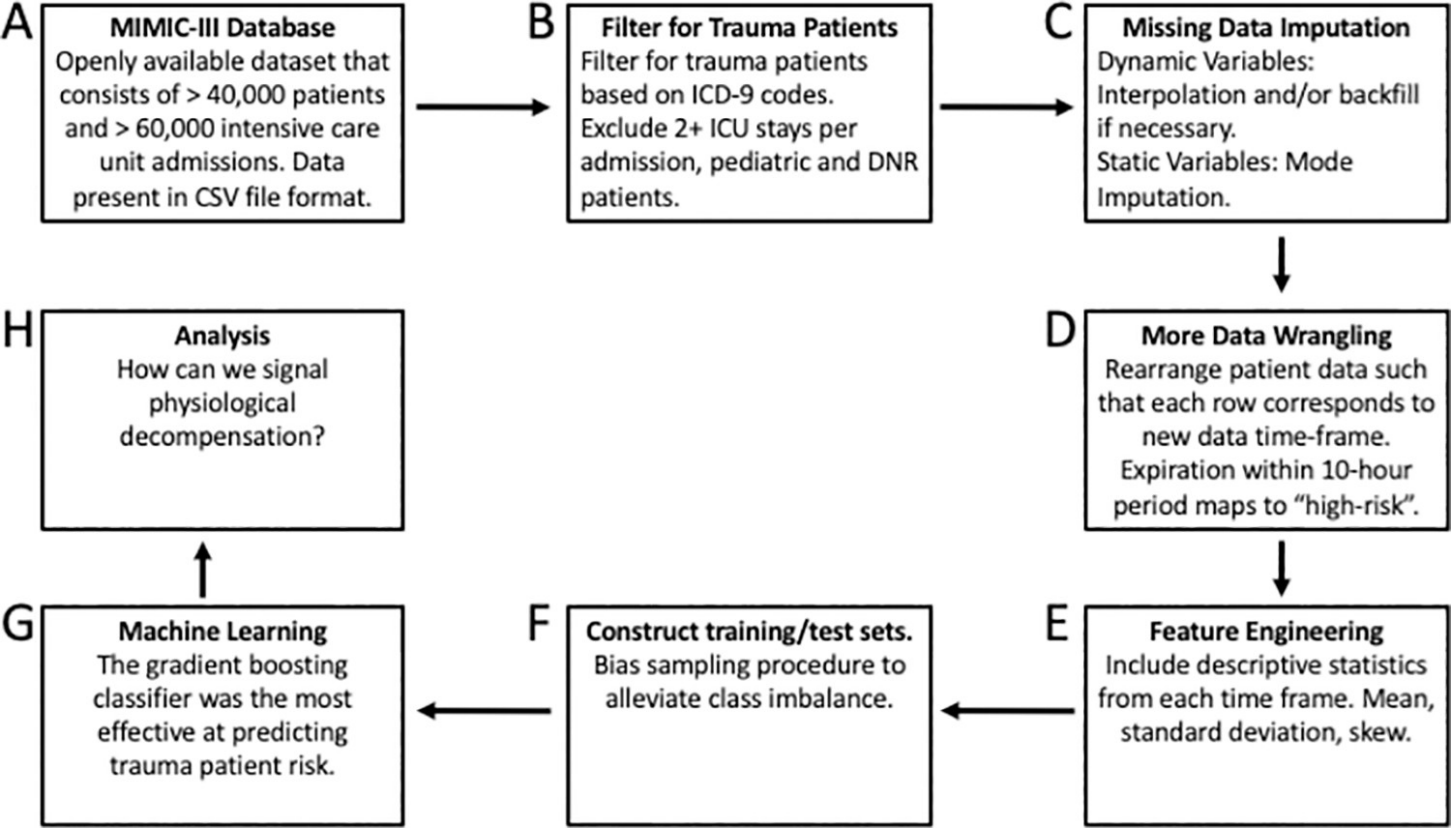
Process flow diagram of the workflow for building a model to predict physiological decompensation. The data comes from the MIMIC-III database, an openly available dataset consisting of >40,000 patients, >60,000 intensive care unit admissions, and 25.csv files. We filtered for trauma patients based on ICD-9 codes, and imputed missing data using mode imputation for static variables and interpolation for dynamic variables. We rearranged the data and posed it as a classification problem within a moving-window, where we tried to predict high-risk (death within 10-hours or death within the final 10% of the patient’s total ICU stay duration). We extracted the mean, standard deviation, and skew for every dynamic variable. We constructed the training and test sets while biasing the sampling procedure to alleviate the class imbalance problem (the ratio of patients who survived to those who expired was ~10:1). We then trained the gradient boosting classifier and analyzed whether it can be used to signal extreme risk of death.

The MIMIC-III dataset is publicly available and consists of more than 60,000 ICU admissions in 25.*csv* files (**Fig 1A**). The patient dataset contains both static features (age, gender, etc.) and dynamic features (heart rate, blood pressure, etc.) and is suitable for the study of time-varying processes in trauma patients. We used an exclusion criterion consistent with the work of Alistair et al., where patients were excluded if they were neonatal or pediatric patients (age < 16), presented in the ICU for less than 4 hours, or had a do not resuscitate order [8]. We also excluded patients with more than a single ICU stay per admission and filtered for patients who had external traumatic injury ICD-9 codes to ensure that we were gathering trauma patients; filtering criteria are listed in **Fig 1B**. Following these exclusion and inclusion criteria, the dataset contained 5400 unique ICU visits (corresponding to 5400 unique patient records). Patient data was recorded at 1-hour time points after admission into the ICU. A table of descriptive statistics describing the patient data included in the analysis is shown in Table 1.

**Table 1:** Descriptive statistics of the trauma patients included in this analysis.

Demographic	Mean +/- S.D.
Age (years)	56.5 ± 20.1
Male fraction	0.61
Height (cm)	171.7 ± 6.7
Weight (kg)	78.0 ± 17.0
BMI (kg/m^2^)	26.9 ± 3.6

### 2.2 Missing data

**Fig 2:**
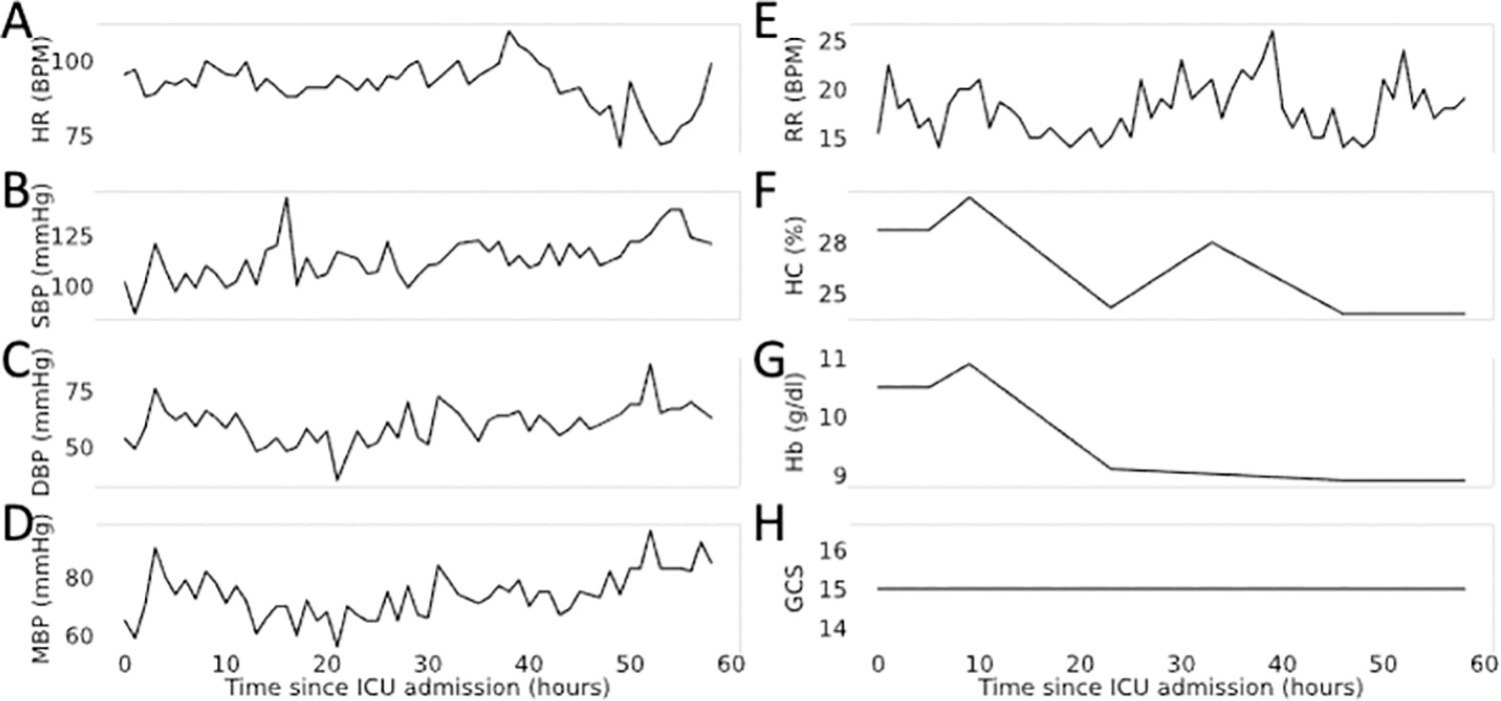
The vital signs of a single patient’s stay in the ICU.

Missing data was handled in one of two ways. Mode imputation was used to impute the missing values and outlier values for all static variables used in the analysis (e.g., age, height, weight, BMI). For dynamic variables, we used linear interpolation method to populate the missing values. The vital signs of an exemplary patient are shown in **Fig 2**. In some cases, the features varied greatly and displayed large fluctuations over the course of a patient’s stay in the ICU (e.g., heart rate, respiratory rate, and systolic, diastolic, and mean blood pressure) and in other cases a variable remained almost constant (e.g., hematocrit, hemoglobin, and Glasgow Coma Score). The high-dimensionality and heterogeneity of such data can become challenging even for an expert to rapidly interpret. Ideally, machine learning models are equipped to accommodate these high-dimensional patterns and use them to predict patient-risk. The rate of missingness for dynamic variables prior to imputation is shown in Table 2. It is important to note that vital signs are recorded every hour while many other dynamic properties are recorded on an as-needed basis. In many instances, the properties are not truly missing they were simply not recorded as frequently as the vital signs.

**Table 2:** Rate of missingness for dynamic variables prior to imputation.

Property	Percent Missing
Heartrate	6.45
Sysbp	10.96
Diasbp	10.97
Tempc	69.11
spo2	8.840
glucose	78.34
endotrachflag	66.35
aniongap	93.98
albumin	99.20
bicarbonate	93.92
bilirubin	98.96
calcium	97.38
creatinine	93.94
chloride	93.5
hematocrit	92.49
hemoglobin	94.31
lactate	97.53
platelet	94.45
potassium	92.16
Ptt	96.39
Inr	96.52
sodium	93.03
Bun	93.95
wbc	94.61

Note that vital signs are recorded more frequently than most other dynamic properties.

### 2.3 Data formatting

**Fig 3:**
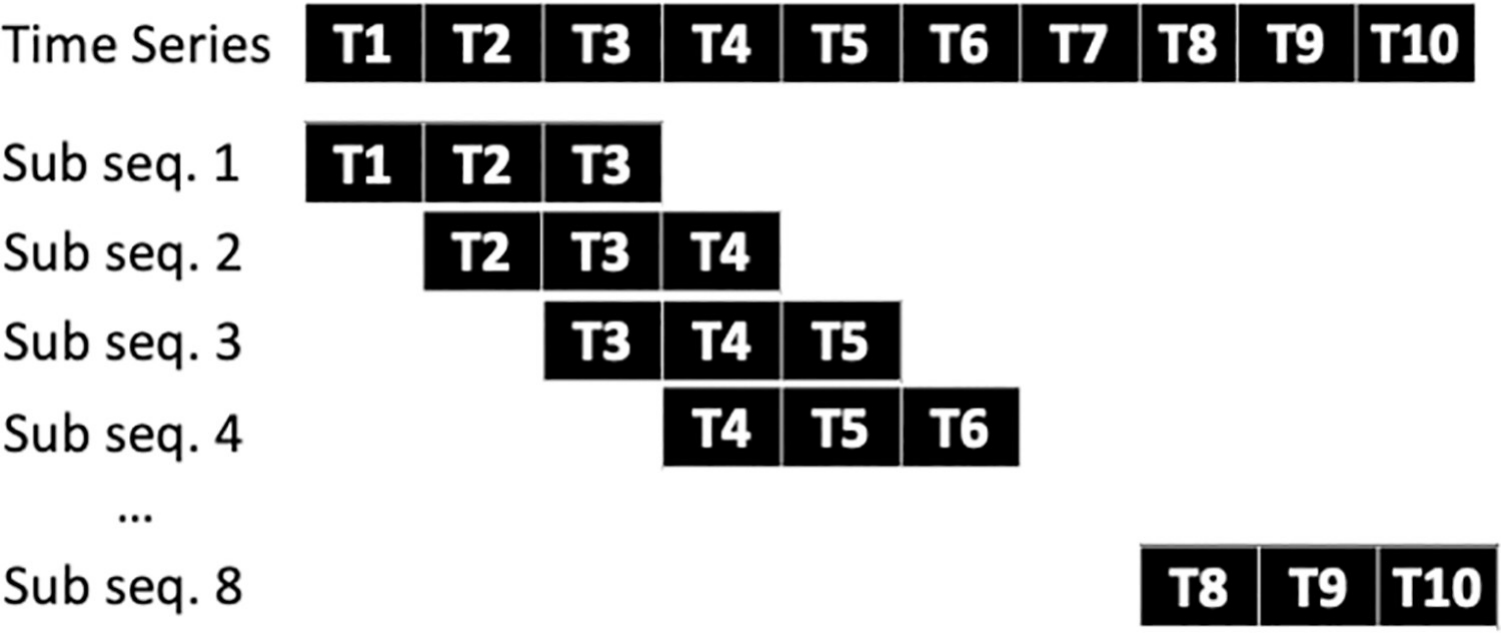
Construction of the dataset from the raw data. Patient trajectories were divided into 3-hour window subsequences (Sub seq.) with a 1-hour step size.

We posed this problem as a binary-classification problem where the data from the 3-previous time points (spaced at 1-hour intervals) was used to predict mortality over the following 10 hours (Fig 3). By posing the problem in this manner, the labels we were seeking to predict were effectively “high-risk” and “low-risk” (**Fig 1D**). We further hypothesized that the most recent patient history was more predictive of patient-risk than the long-term patient history. Since we computed the descriptive statistics of each of the features, this would be akin to computing these statistics with an exponential moving average and a small half-life (thus, increasing the weight of the most recent data points).

### 2.4 Feature selection

The dataset consisted of both static and dynamic variables. A common approach to the treatment of dynamic patient data is to extract features based upon the entire duration of a patient’s stay. In this analysis, we performed a different method where we divide the original patient vital sign evolution into multiple subsequences with a fixed step size and window length. This approach implicitly assumes that the short-term patient history is more significant to the outcome of the patient than the long-term patient history. For each trauma patient, we specified a 3-hour time window referred to as a ***patient time fragment***. Within each time fragment, we extracted the mean, standard deviation, and skew for each of the dynamic variables, as shown in **Fig 1E**.

Importantly, only vital sign and lab measurements from the basic metabolic panel were used and a complete list of features is shown in Table 3. This is consistent with the work of Alistair et al. who used similar features in their analysis [8]. In addition to vital sign and lab measurement data, we also included the survival probability prediction of the model from our previous publication based only on time-equals-zero admission data [10]. While the model from our previous publication (PLOS model) had been trained on static data taken primarily from trauma patients upon admission to the hospital, the inputs to this model (age, gender, temperature, GCS, SBP, O2SAT, HR, RR) are all present in this MIMIC-III dataset. Therefore, the survival probability predictions from the PLOS model were calculated and included as a feature for each patient at every time-point. We also included the static variables shown in the second column of Table 3, which were held constant across all patient time fragments. This analysis resulted in 89 features (5 static variables, and 3 features extracted from each of the 28 dynamic variables).

**Table 3:** Features extracted from the dataset.

Dynamic Variables	Static Variables
Heart Rate	Sex
Systolic Blood Pressure Diastolic Blood Pressure Mean Blood Pressure Respiratory Rate Temperature Oxygen Saturation Glucose levels Glasgow Coma Score Anion Gap Albumin levels Bicarbonate levels Bilirubin levels Calcium levels Creatine levels Chloride levels Lactate levels Platelet levels Potassium levels Sodium levels Prothrombin Time International Normalized Ratio Hematocrit Hemoglobin Blood Urea Nitrogen White Blood Cell count Endotracheal tube requirement flag PLOS-NTDB Model Prediction (10)	Age Height Weight BMI

Importantly, these extractions occurred over a 3-hour window of time length for each patient. A 1-hour step size was used to construct each patient fragment. The mean, standard deviation, and skew was included for each of the dynamic variables.

### 2.5 Class imbalance

Of the 5400 trauma patients in the MIMIC-III database, 526 of them died. While not an enormous class imbalance, the dataset consisted of 158,108 patient windows of which only 5,615 mapped to high-risk patients. If one were to randomly assign patients to either the training set or the test set without accounting for this imbalance, the model will simply learn to label each patient as low-risk (the majority class) as this will maximize its accuracy (although it would not have learned anything meaningful). To alleviate class imbalance, we undersampled from the population that survived to develop the training set, as described in **Fig 1F** [19]. Patients who ultimately died were assigned a higher probability of being placed in the training set than in the test set. Importantly, patients were marked to be placed in either the training set or the test set to prevent data leakage.

### 2.6 Machine learning modeling

A gradient boosting classifier was trained to predict high-risk and low-risk patient windows, the penultimate step in the process flow diagram and shown in **Fig 1G** [20]. The algorithm works by training 300 weak learners, typically short decision trees, on the dataset in a stage-wise manner so that the errors made by the early weak learners are corrected by the latter ones. Gradient boosting receives high recognition in the literature for its effectiveness in classification on tabular data [21,22]. Hyperparameter selection was performed via grid search cross validation, a method by which combinations of hyperparameters are selected for the model and validated against withheld samples of the training set to approximate how well they will generalize to the test set. We also trained other common machine learning models to the dataset (e.g., support vector machine, logistic regression, recurrent neural network, and gaussian naïve Bayes) but the gradient boosting classifier was found to outperform all other models. With the model trained, we were left to interpret the results as represented in **Fig 1H**.

## 3. Results

**Fig 4:**
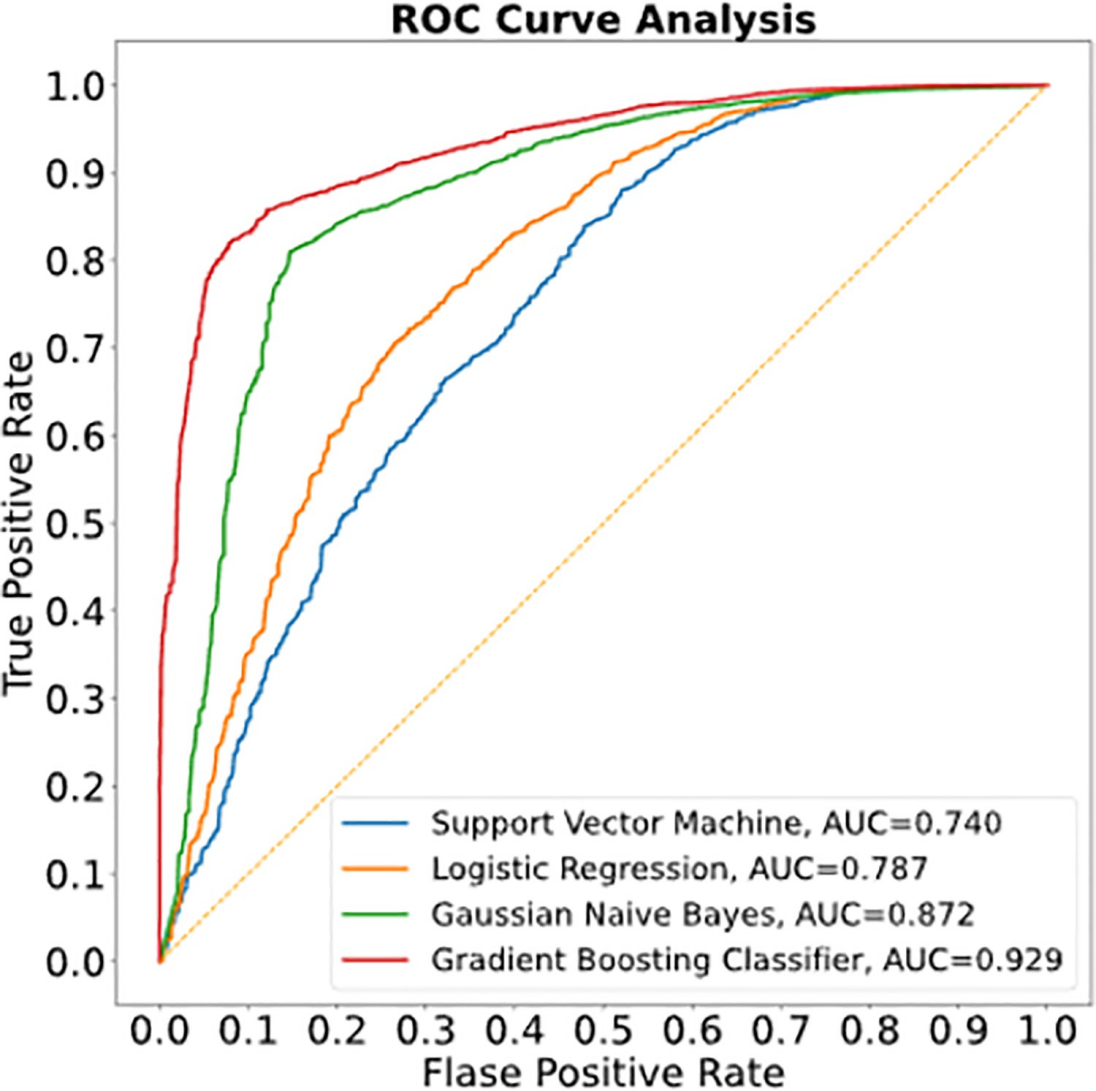
The receiver operator characteristic curve for each machine learning model considered in the present study. The gradient boosting classifier is the most accurate and the one used in the subsequent analysis.

The accuracy of the model was expressed in terms of the area under the receiver operator characteristic curve (AUROC), which is the probability of correctly distinguishing between a high-risk patient time fragment and a low-risk patient time fragment. Using this as the metric for the model, we achieved an accuracy of 92.9%. We also compared this AUROC with other machine learning models and found that it outperformed the support vector machine classifier, the logistic regression classifier, and the Gaussian Naïve Bayes classifier (Fig 4). A recurrent neural network was implemented to explicitly model the long-term relations, but the RNN overfit the training set (AUROC = 0.79).

**Fig 5:**
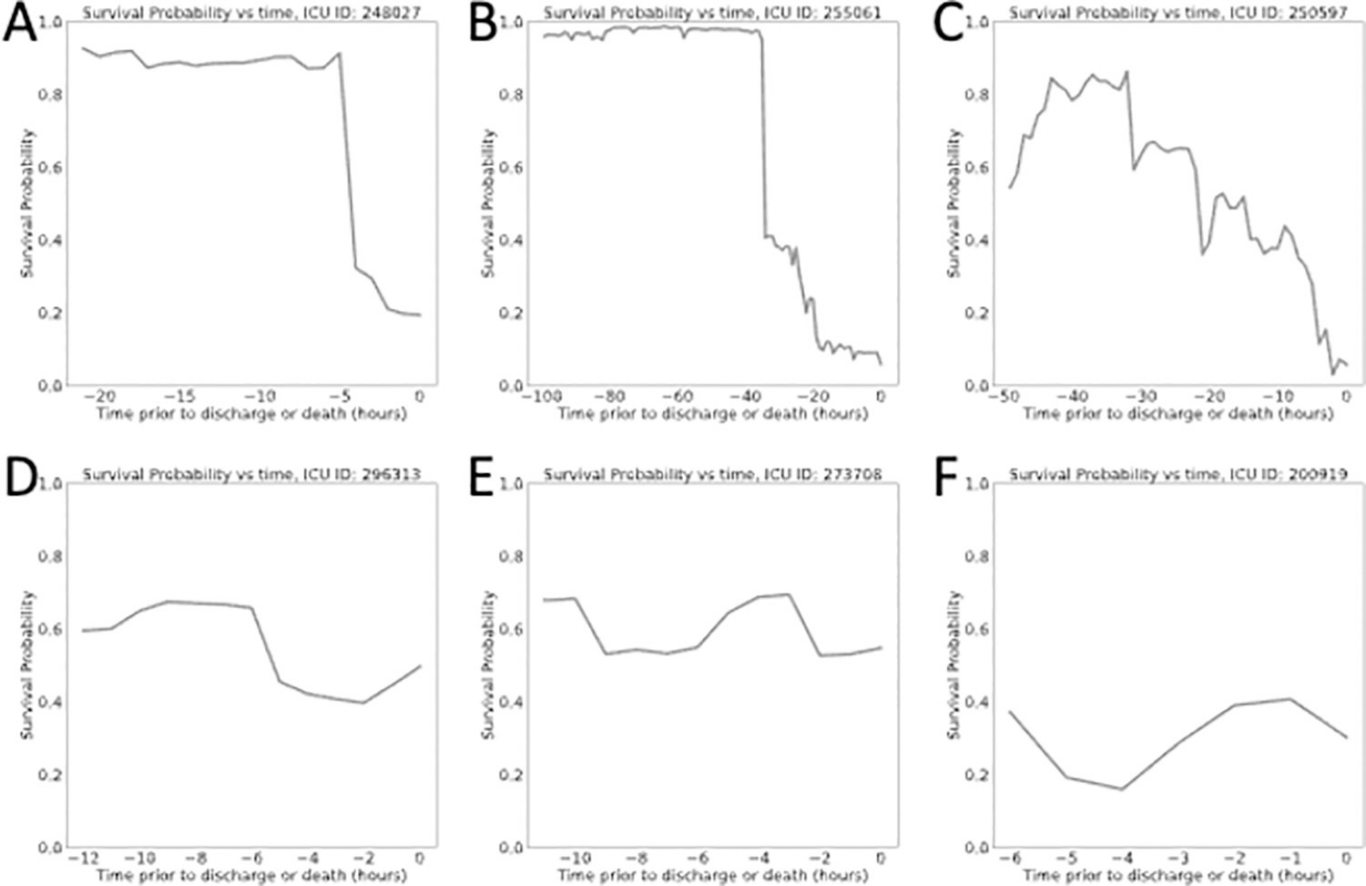
Survival probability plots for a subset of patients who ultimately expired. Notice that many of these patients experienced a sudden dip in survival probability prior to death, except for one displayed in panel F, where the probability of survival of this patient was consistently low (<0.4 for all time points).

**Fig 6:**
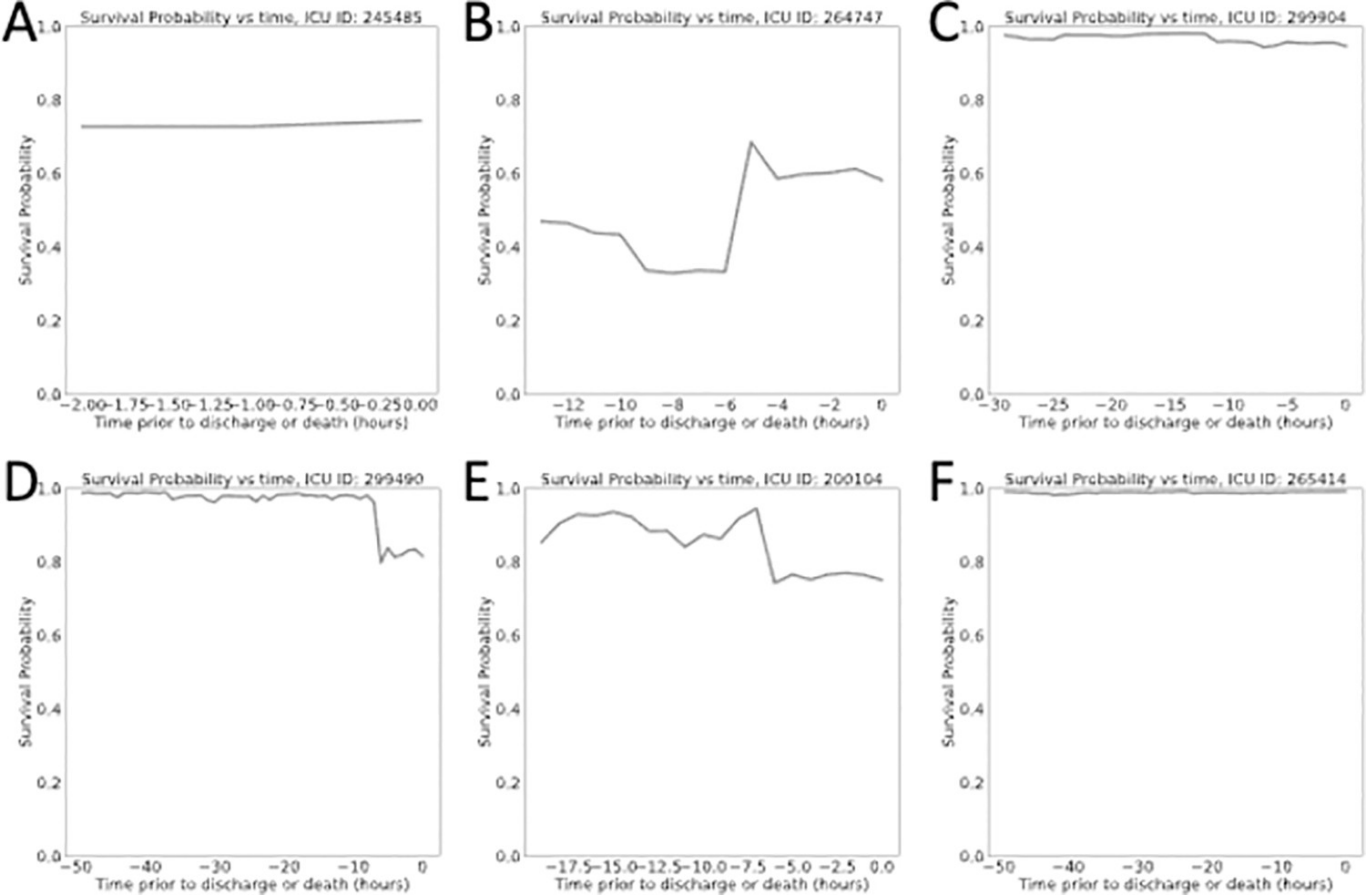
Survival probability plots for a subset of patients who survived. Note that probability of survival remains the same or increases, but rarely displayed a sudden, dramatic drop.

Probability of survival plots were computed by applying the model every hour once the patient had accumulated a minimum of 3-hours in the ICU. We plotted the probability of survival against the time prior to expiration for patients who died (Fig 5) and prior to discharge for those who survived (Fig 6). The survival probability plots of patients who died exhibited either a sudden drop in survival probability in the hours prior to death or had survival probabilities weakly fluctuating at very low survival probability values (e.g., **Fig 5E and 5F**). In the patients who survived, we generally observed the opposite behavior. Survival probabilities would either rise, remain the same, or experience minor drops that were not indicative of extreme risk of death.

**Fig 7:**
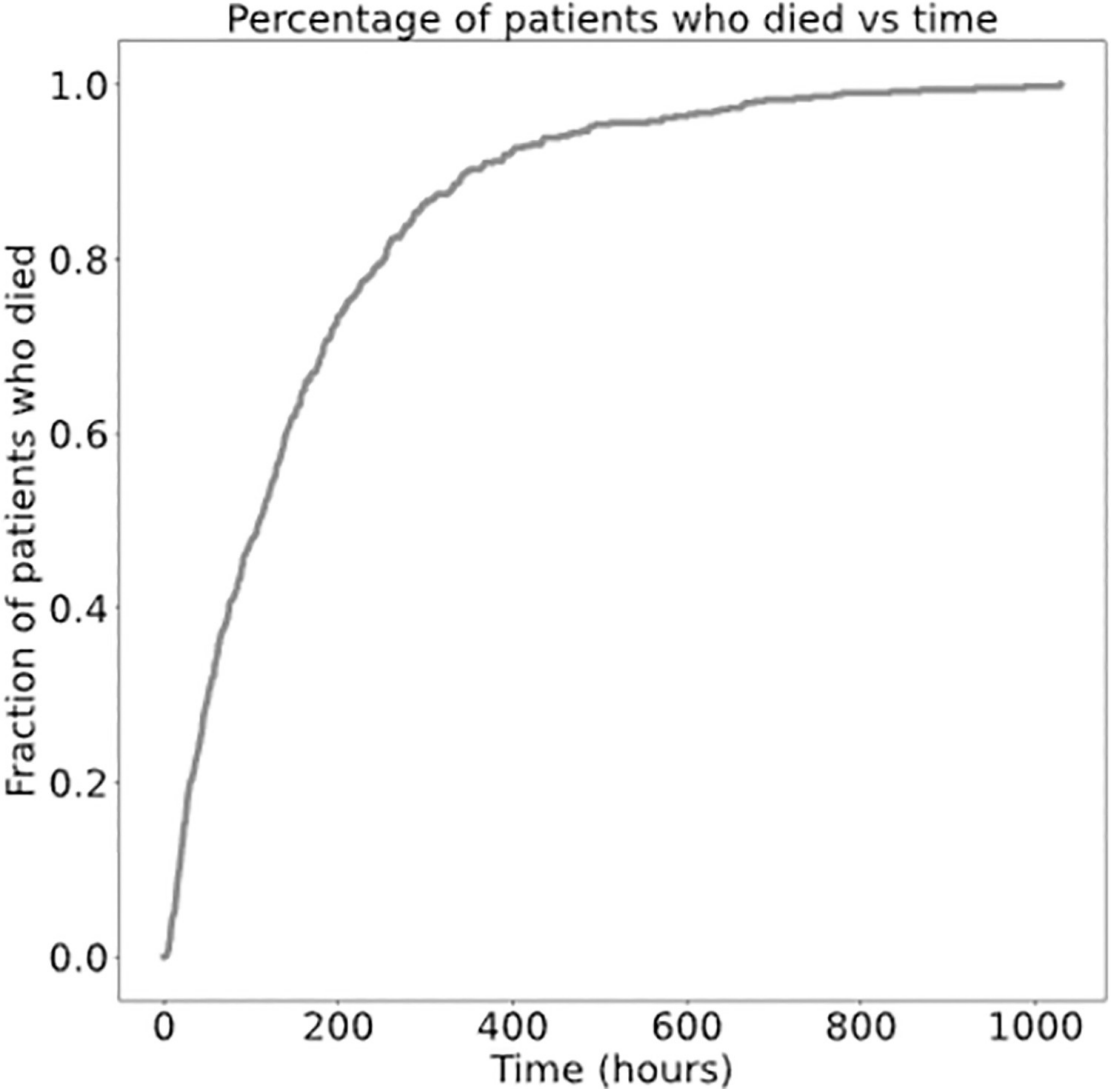
A plot of fraction of patients who died against time. The majority of patients die within the first 72 hours again showing that the sooner the patient can receive treatment the greater the likelihood of survival.

We also plotted the fraction of patients who died as a function of time (Fig 7). We can see that about 20% of trauma patients who die do so within the first 24 hours of trauma and 50% within the first 72 hours. This is consistent with the literature, as it well-established that treating a trauma patient in the early stages of injury is vital to increasing survival probability.

## 4. Discussion

The gradient boosting classifier was able to accurately predict risk in the trauma patients present in the MIMIC-III database with dynamic (vital signs, basic metabolic panel values) and static (age, gender, etc.) values. This machine learning methodology naturally raises certain questions as to how the model should be implemented in a clinical setting and interpreted mechanistically. If an absolute, and arbitrary, threshold is used to indicate extreme risk of death, it would not be accurate for detecting this extreme risk in severe cases. An outlier detection method for detecting sudden drops in survival probabilities may be more appropriate, as it will be able to trigger a response from ICU team. As it stands, the relatively small number of deceased patients make it difficult to determine the model’s effectiveness, but as more data becomes available, this should be the focus of future work.

**Fig 8:**
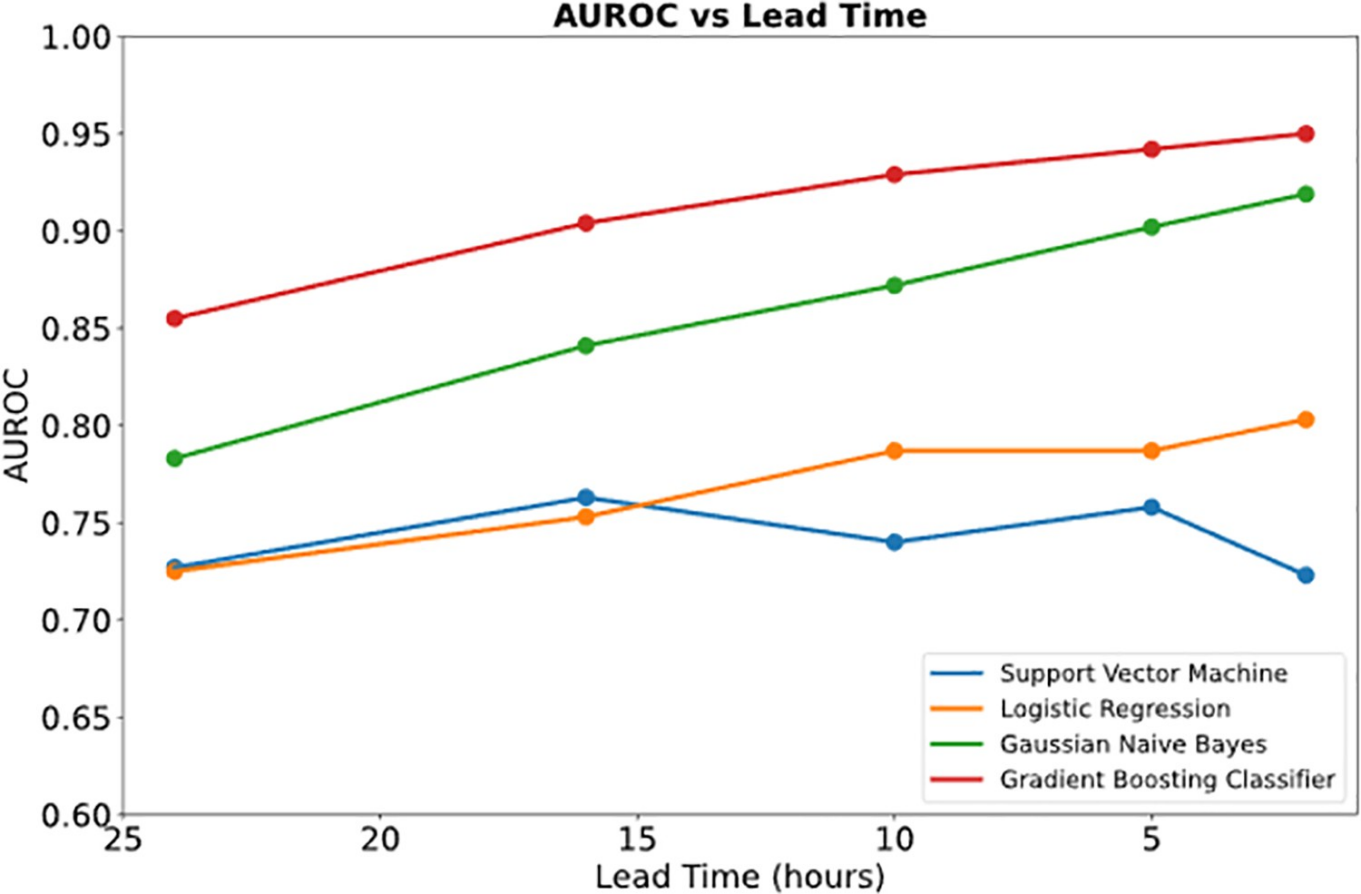
A sensitivity analysis of the lead time for each of the 4 machine-learning models. A 10-hour lead-time achieves a good compromise between high AUROC and sufficient lead time for action to be taken by clinical staff. The gradient boosting classifier outperformed each of the other 3 models regardless of the label start time.

One of the key assumptions of this analysis was that the time prior to death or discharge from the ICU was specified prior to training, a variable we will refer to as lead-time. We performed a sensitivity analysis on this variable, as it represents a balance between predictive power (high AUROC) and value in a clinical setting (the greater the lead-time, the more useful the model is) which is shown in Fig 8. Based on the concavity of plot and apparent inflexion point around 10-hours, this value seems to be a fair compromise between these two factors.

There are other limitations of the model presented in this work. First, all data used in this analysis are taken from the same hospital making it unclear if it is predictive of all hospitals. In theory, a similar approach can be taken if data is unified from participating trauma centers but to our knowledge this has not yet been carried out. The National Trauma Data Bank (NTDB) consists of trauma patient data from multiple centers but is mostly restricted to static data [23]. When the PLOS-NTDB model (trained only with NTDB data) was used as time-dependent sole predictor for model accuracy with the MIMIC-III data set, we achieve an AUROC of 0.70. While this is low, the PLOS-NTDB model was greater than an AUROC of 0.50, indicating that it was still a useful feature that should be included. Second, our analysis was only performed on trauma patients suggesting that it may not generalize to other disease states. Furthermore, while our data processing methodology in 158,108 unique time points, this only represented 5,400 trauma patients, a relatively small number. As is usually the case in problems with limited data available, more data could dramatically improve AUROC and garner more insights. We hope that this paper, as well as the studies of other groups, can help elucidate the need for publicly available, de-identified, patient data for future in-depth analyses.

Another limitation of the present model is its interpretability. While gradient boosting classifiers tend to perform very well on tabular datasets, they often lack the interpretability of simpler models such as the logistic regression classifier. This is a substantial drawback because clinicians must have an understanding of how a model works in order to trust it. Tools such as the Local Interpretable Model-Agnostic Explanations method (LIME) or Shapley Additive Explanations (SHAP) value metrics are used to interpret these models, but their reliability remains an active area of research [24]. Interpretability is a key component of a machine learning models in most settings, but especially in healthcare since it may often have the direst consequences. One way to interpret the model is to examine the coefficients of the logistic regression model in the same way one would in a multivariate linear regression model–the magnitude of the coefficients is a metric for feature importance. The largest coefficients correspond to the mean vital signs (mean blood pressure, respiratory rate, temperature, oxygen saturation, and gcs), the mean and standard deviations of many of the basic metabolic panel features (glucose, aniongap, albumin, bilirubin, calcium, creatine, chloride, hematocrit, hemoglobin, lactate, platelet, and wbc), and the patients’ BMI. Intuitively, this is consistent with expectation. The mean of the vital signs and the standard deviation (an indicator of the variability of these variables), should be strong indicators of patient health.

Clearly, publicly available EHR spell out data presents an opportunity for patient outcome improvements. In this work, we demonstrated the utility of gradient booster classification in handling static and dynamic data. Additionally, we demonstrated the utility of the patient time fragment as a useful protocol for extracting information from time series with the goal of training machine learning algorithms. Importantly, the dynamic survival probability plots for patients who die appear to be considerably different from those who survive, an example of the benefit of reducing the high dimensionality of the patient record to a single trauma trajectory.
